# Tooth discoloration induced by apical plugs with hydraulic calcium silicate-based cements in teeth with open apices—a 2-year in vitro study

**DOI:** 10.1007/s00784-021-04009-0

**Published:** 2021-06-21

**Authors:** Ralf Krug, C. Ortmann, S. Reich, B. Hahn, G. Krastl, S. Soliman

**Affiliations:** 1 Department of Conservative Dentistry and Periodontology and Center of Dental Traumatology, University Hospital of Würzburg, Pleicherwall 2, 97070 Würzburg, Germany; 2 Radolfzell, Germany; 3 Eichstätt, Germany

**Keywords:** Mineral trioxide aggregate, Portland cement, Tooth discoloration, Spectrophotometer, Blood contamination, Dentin sealing

## Abstract

**Objectives:**

To assess tooth discoloration induced by different hydraulic calcium silicate-based cements (HCSCs), including effects of blood and placement method.

**Materials and methods:**

Eighty bovine teeth cut to a length of 18 mm (crown 8 mm, root 10 mm) were randomly assigned to 10 groups (*n* = 8), receiving orthograde apical plug treatment (APT). Apical plugs were 4 mm in length and made of ProRoot MTA (Dentsply), Medcem MTA (Medcem), TotalFill BC RRM Fast Set Putty (Brasseler), or Medcem Medical Portland Cement (Medcem) plus bismuth oxide (Bi2O3) with and without bovine blood. Further, orthograde (with or without preoperative adhesive coronal dentin sealing) and retrograde APT were compared. Teeth were obturated with gutta-percha and sealer, sealed with composite and stored in distilled water. Tooth color was measured on apical plug, gutta-percha/sealer, and crown surface before treatment versus 24 h, 1, 3, 6, 12, and 24 months after treatment by spectrophotometry. Color difference (ΔE) values were calculated and analyzed by Shapiro–Wilk test, ANOVA with post hoc tests, Friedman test, *t* test, and post hoc tests with Bonferroni correction (α = .05).

**Results:**

Tooth discoloration occurred in all groups with no significant differences between HCSCs (*p* > .05). After 24 months, color changes were prominent on roots but insignificant on crowns. Blood contamination induced a significantly decreased luminescence (*p* < .05). Blood had a stronger impact on tooth color than Bi_2_O_3_. No relevant effects of retrograde placement (*p* > .05) or preoperative dentin sealing (*p* > .05) were detected.

**Conclusions:**

Apical plugs of the tested HCSCs cause discoloration of bovine roots, but not discoloration of bovine tooth crowns within a 24-month period.

**Clinical relevance:**

APT should be performed carefully while avoiding direct contact with the coronal dentin, and in that case no aesthetic impairments occur.

## Introduction

Endodontic treatment of teeth with incomplete root development and thin dentinal walls is often challenging and prone to complications. To overcome major drawbacks of conventional apexification such as cervical root fracture [1], hydraulic calcium silicate-based cements (HCSCs), e.g., mineral trioxide aggregate (MTA), were introduced as state-of-the-art root-end closure materials that can be placed during single-visit apexification. Clinical outcomes obtained with this approach were similar or superior to those achieved with long-term calcium hydroxide apexification. In several studies, success rates ranged from 76.5 to 93.5% for teeth with open apices treated with MTA plugs [2–8]. One long-term case series attained a 96% healing rate in nonvital immature permanent anterior teeth 8.3 years after treatment with MTA plugs [9].

HCSCs are known to induce a broad potential of tooth discoloration. Many laboratory studies have shown dentinal discoloration of varying severity, especially when HCSCs come in contact with blood or sodium hypochlorite solution [10–14]. Further effects of radiopacifiers, light, oxygen, pH, and mixing agents have also been discussed [14–17]. Changes in tooth color most commonly occurred a few days after placement of these materials. There is a clinical evidence of coronal tooth discoloration associated with MTA and Biodentine coronal plugs in non-vital immature teeth when performing revascularization [18]. However, for conventional endodontic treatment, there is scarce evidence regarding a relevant coronal discoloration potential causing aesthetic impairment after HCSC placement within the root canal [19].

In the context of dental trauma, anterior immature teeth of children and young adolescents are often those in need of endodontic interventions with HCSC apical plugs. The material must be placed cautiously within the root close to the apical foramen, obviously far away from the aesthetically relevant tooth crown. The known manifestation of tooth discoloration caused by HCSC varies heterogeneously, depending on factors such as the technique of HCSC placement, irrigants, storage conditions, measuring devices, duration, and the specific type of endodontic material and its composition [20]. Studies have shown that substantial discoloration of white MTA and bismuth oxide (Bi2O3) radiopacifiers occurs after contact with sodium hypochlorite solution [11, 14] and that the presence of blood adjacent to setting white MTA/HCSC exacerbates the discoloration [12, 13, 21, 22]. Two studies showed that the use of zirconium oxide as the radiopacifier agent did not lead to discoloration of dental hard tissue [23, 24]. Furthermore, it was reported that discoloration of the dental hard tissue can be avoided by using tantalum oxide as the radiopacifier [25]. Clinical investigations observed aesthetic impairment after the placement of HCSC apical plugs [9, 26]. Therefore, it is assumed that sealing the pulp chamber walls with a dentin bonding agent might decrease HCSC-related discoloration [27, 28].

To the best of the authors’ knowledge, neither the effects of HCSC apical plugs on coronal tooth discoloration nor the interaction of HCSC apical plugs with blood have been investigated previously. It is still unknown if such apical plug materials affect the color of the tooth crown and, thus, impair aesthetics.

This in vitro study aimed to investigate the tooth discoloration potential of different HCSCs used for apical plug treatment (APT) of bovine teeth over a 24-month period while taking the effects of blood, preoperative dentin sealing, and the technique of HCSC placement (orthograde versus retrograde) into account. The null hypothesis stated that the four tested HCSCs do not induce aesthetically relevant tooth discoloration. Three research questions were stated: Does admixed blood increase tooth discoloration? Does the retrograde placement of HCSC reduce coronal tooth discoloration compared with orthograde placement? Does adhesive sealing of the dentin in the pulp chamber (before HCSC placement) reduce tooth discoloration?

## Material and methods

### Specimen preparation

This experimental study was conducted in conformity with the principles set forth in the WMA Statement on Animal Use in Biomedical Research. Eighty bovine incisors from cattle 4–6 years of age were extracted and stored in 1% chloramine-T solution at room temperature. The periodontal ligament of each tooth was removed with scalpels and gauze pads. Teeth were cut to a standard crown length (8 mm) and root length (10 mm) using a diamond saw. Calipers (Karl Hammacher GmbH, Solingen, Germany) were used to measure distances between each cut surface and the most apical point of the cement-enamel junction (CEJ) on the labial side of each tooth. Pulp tissue was removed manually using an ISO 60 stainless steel file (Dentsply Sirona, Bensheim, Germany), and the root canal was subsequently rinsed with distilled water. Root canals were reamed to a standard diameter of 2.3 mm using a bur (410 RFX 023, Busch & Co GmbH, Engelskirchen, Germany). A cylindrical diamond bur (No. 030301, 6 mm diameter, Busch & Co GmbH, Engelskirchen, Germany) was used to flatten the outer vestibular convexity to make a flat plane for the measuring probe of the digital spectrophotometer (VITA Easyshade V; VITA Zahnfabrik AG, Bad Säckingen, Germany) to rest on the tooth surface. Each specimen was stored in distilled water.

Following root canal preparation, endodontic irrigation was performed in an ultrasonic bath: 24 h before root canal obturation, the specimens were immersed in 3% sodium hypochlorite for 30 min, followed by 20% EDTA for 2 min, and 3% sodium hypochlorite for 3 min. Root canals were rinsed with distilled water between each irrigation step.

The following four commercial HCSC products were investigated in the present study:

1. ProRoot MTA (Dentsply Tulsa Dental, Tulsa, United States), the first commercial HCSC product used to perform root-end closure in teeth with wide apices [29–31], contains 75% Portland cement, 5% calcium sulfate dehydrate, and 20% Bi2O3 as the radiopacifer [24].
2. Medcem MTA (Medcem GmbH, Weinfelden, Switzerland), a so-called second generation MTA, consists of Pure Portland Cement® with zirconium oxide as the radiopacifer. It is recommended for perforation repair [32], apexification [33], surgical endodontics [34], pulp capping [35], and pulpotomy [36]. Medcem MTA root-end-fillings have shown low leakage [37].
3. Medcem Medical Portland Cement (MMPC) is recommended for direct and indirect capping of permanent and primary teeth and for pulpotomy, perforation repair, and apexification [38]. Because it contains only Portland cement with no additional ingredients, it is believed that MMPC does not cause tooth discoloration.
4. TotalFill BC RRM Fast Set Putty (Brasseler GmbH, Lemgo, Germany), a high-viscosity modification of the TotalFill BC Sealer, contains only calcium sulfate and tantalum pentoxide instead of calcium hydroxide and should thus have a low discoloration potential.

The specimens were randomly assigned to ten groups of eight specimens each. All teeth were apically filled with one of the four tested HCSCs with or without the admixture of bovine blood, as specified in Table 1. In groups 7 − 10, Bi2O3 was added to Medcem Medical Portland Cement to assess the influence of Bi2O3 on tooth color stability. During the obturation procedure, each specimen was fixed in silicone sockets (Silaplast, Detax GmbH, Ettlingen, Germany). Apical plugs of each HCSC were placed from the apical terminal point using an applicator (Dentsply Sirona, Bensheim, Germany) with a 1.2-mm tip and a dental microscope (Zeiss AG, Jena, Germany) with 0.6 × magnification. The material was condensed with pluggers and paper points to obtain a material thickness of 4 mm. All HCSCs were placed orthograde except in group 9. Efforts were taken to ensure that the coronal cavity was not contaminated during orthograde placement. All dentinal walls were cleaned using microbrushes soaked in distilled water after HCSC placement. The remaining part of the root canal was filled with sealer (AH plus, Dentsply Sirona, Bensheim, Germany) and warm gutta-percha up to 1 mm below the CEJ using a squirting technique. The cavity was cleaned with cotton pellets moistened with 70% ethanol.

**Table 1:** Groups of specific test materials and mixing ratios used for apical plug treatment (APT) with ProRoot MTA (PRMTA), Medcem MTA (MMTA), or TotalFill BC RRM Fast Set Putty (TF) with and without bovine blood (B) and for retrograde and orthograde APT with Medcem Medical Portland Cement (MPC) plus Bi2O3 (Bi) with and without bovine blood (B) after adhesive sealing (Adh) of the coronal dentin using ScotchBond Universal Adhesive (3 M Espe, Landsberg/Lech, Germany)

Group (G)	Material	Cement [mg]	Bi_2_O_3_ [mg]	Distilled water [µl]	Bovine blood [µl]
G01-PRMTA	ProRoot® MTA	181	0	50	0
G02-PRMTA-B	ProRoot® MTA + bovine blood	181	0	33	17
G03-MMTA	Medcem MTA	181	0	50	0
G04-MMTA-B	Medcem MTA + bovine blood	181	0	33	17
G05-TF	TotalFill BC RRM Fast Set Putty	181	0	0	0
G06-TF-B	TotalFill BC RRM Fast Set Putty + bovine blood	181	0	0	17
G07-MPC-Bi	Medcem Medical Portland cement + Bi_2_O_3_	145	36	50	0
G08-MPC-Bi-B	Medcem Medical Portland cement + Bi_2_O_3_ + bovine blood	145	36	33	17
G09-MPC-Bi-Retro-B	Medcem Medical Portland cement + Bi_2_O_3_ + bovine blood + retrograde placement	145	36	33	17
G10-MPC-Bi-Adh-B	Medcem Medical Portland cement + Bi_2_O_3_ + bovine blood + adhesive sealing (Adh)	145	36	33	17

In group 10, the dentin was adhesively sealed within the coronal cavity down to 1 mm below the CEJ using a universal bonding agent (ScotchBond Universal Adhesive, 3 M Espe AG, Landsberg/Lech, Germany) before APT. This seal was subsequently removed with a diamond bur after the apical plug and the remaining part of the root canal filling had set. In group 9, the entire root canal was first filled with gutta-percha and sealer, and, afterwards, 4 mm of the apical part of the root canal filling was removed via the apical foramen using heat pluggers and then filled with a mixture of Medcem Medical Portland Cement, Bi2O3, and bovine blood.

**Fig. 1:**
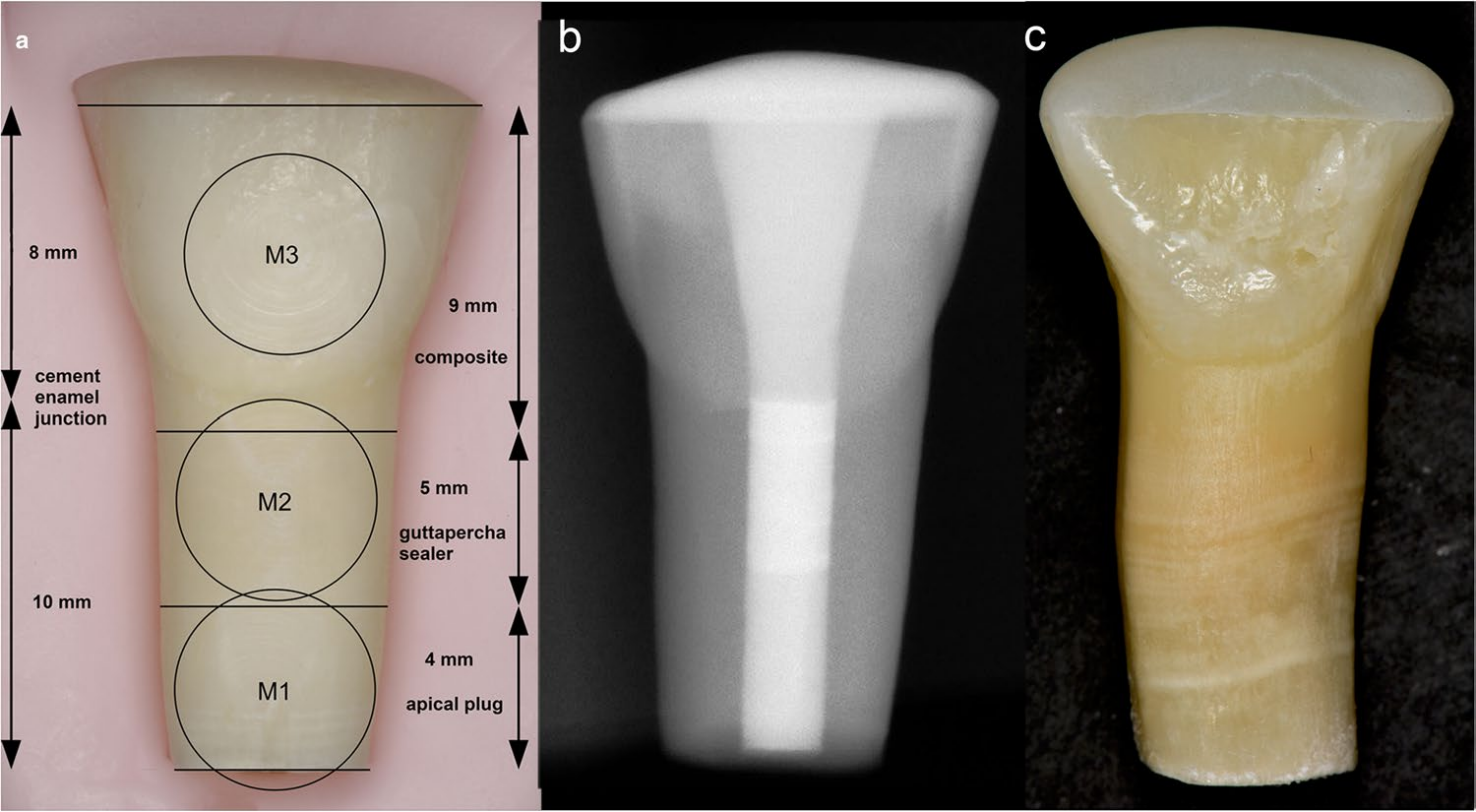
**a** Representation of the three measuring sites: center of the hydraulic calcium silicate-based cement (HCSC) apical plug (M1), middle of root canal filling, consisting of gutta-percha and sealer without any apical plug material (M2), and center of labial crown surface (M3). **b** Radiograph showing the apical plug made of HCSC, the middle of the root canal filled with gutta-percha and sealer, and the coronal cavity filled with composite. **c** Representative specimen (HCSC plug admixed with blood) showing an apical plug treatment (APT) induced discoloration on the apical root surface

Finally, all coronal cavities were filled with a translucent flowable composite (SDR, Dentsply Sirona, Bensheim, Germany) after etching the enamel with 37% phosphoric acid and applying a universal bonding agent. Finally, light curing was performed for 20 s using an LED curing light (EliparTM Free Light 2, 3 M Espe AG, Landsberg/Lech, Germany). Each tooth was stored individually in a labeled tube with distilled water at a temperature of 37° C (Figs. 1a − c).

### Color determination

Color measurement was performed under standardized illumination conditions with a calibrated spectrophotometer (VITA Easyshade®V, VITA Zahnfabrik AG, Bad Säckingen, Germany) and data from the L*a*b System (Commission Internationale de l’Eclairage). Each tooth specimen was placed in an individually molded cradle made of silicon, which served as the specimen holder. The values of change in color (ΔE) always represent a change between two color readings. Color measurements were taken before root canal filling (T0), 24 h post-treatment (T1 = baseline), and 1 (T2), 3 (T3), 6 (T4), 12 (T5), and 24°months (T6) after filling placement. All readings were repeated three times and then averaged. In the present study setting, measurements of a negative (empty sample) and a positive (sample with root canal filling without any apical plug) group could be excluded. On the one hand, the color measurements using the L*a*b System require values of a quantifiable change in color (ΔE), which cannot be obtained in case of an empty sample. On the other hand, assessing the potential effect of apical plug induced discoloration in the aesthetic zone required the presence of HCSCs in order to answer the stated research questions.

The change in color (∆E) of the bovine tooth specimens caused by HCSCs was calculated as the color difference between the empty root canal before treatment (T0; baseline control) and the filled root canal 24°h after filling (T1) (Table 2) as well as between T1 (24°h) and T6 (24°months) for each HCSC test group, as determined by spectrophotometry (Table 3). By this approach, the effect of degradation of the materials inside the root canal can be assessed independently of the effect of the material’s own color. Color difference (∆E*_ab_), here also denoted as ∆E, was calculated as a function of the change in luminescence (∆L) with L* for the lightness from black (0) to white (100), a* from green ( −) to red ( +), and b* from blue ( −) to yellow ( +), as described below (Eq. 1).

1 $$\begin{array}{c}{\Delta E}_{ab}^{*}= \sqrt{\Delta {L}^{*2}+ \Delta {a}^{*2}+ \Delta {b}^{*2}}=\\ =\sqrt{{({L}_{1}^{*}-{L}_{0}^{*})}^{2}+{{(a}_{1}^{*}-{a}_{0}^{*})}^{2}+{{(b}_{1}^{*}-{b}_{0}^{*})}^{2}}\end{array}$$

**Table 2:** Median color change (∆E) with interquartile range (IQR) from pre-treatment (T0) to 24 h post-treatment (T1) on the apical plug (M1), gutta-percha/sealer (M2), and tooth crown/resin composite (M3) surfaces of all groups

	Root		Crown
Group	Median ∆E (IQR) M1	Median ∆E (IQR) M2	Median ∆E (IQR) M3
G01-PRMTA	5.2 (4.1–9.6)	5.3 (4.4–6.0)	1.4 (0.8–2.9)
G02-PRMTA-B	10.8 (8.3–16.3)	6.3 (3.8–12.5)	1.9 (1.1–2.3)
G03-MMTA	7.2 (5.6–9.6)	5.7 (3.6–10.4)	3.4 (2.5–4.0)
G04-MMTA-B	11.2 (8.0–13.5)	9.6 (6.4–13.1)	1.4 (1.3–1.6)
G05-TF	5.4 (4.1–6.9)	4.5 (3.8–5.4)	2.7 (1.1–4.0)
G06-TF-B	6.2 (4.2–7.7)	4.2 (4.0–4.5)	2.2 (1.2–3.3)
G07-MPC-Bi	5.4 (4.6–8.1)	5.6 (4.4–8.1)	2.4 (1.8–4.0)
G08-MPC-Bi-B	11.3 (8.1–17.2)	8.2 (3.8–11.2)	1.7 (1.1–2.8)
G09-MPC-Bi-Retro-B	8.5 (6.9–9.5)	5.2 (4.5–7.2)	1.6 (1.4–2.2)
G10-MPC-Bi-Adh-B	11.6 (10.0–14.4)	5.5 (4.1–7.7)	3.3 (2.5–4.6)
All groups pooled	8.3 (5.4–11.0)	5.3 (4.1–8.2)	2.1 (1.4–3.2)

**Table 3:** Median color change (∆E) with interquartile range (IQR) from 24 h (T1) to 24 months post-treatment (T6) on the apical plug (M1), gutta-percha/sealer (M2), and tooth crown/resin composite surfaces (M3) of all groups

	Root		Crown
Group	Median ∆E (IQR) M 1	Median ∆E (IQR) M 2	Median ∆E (IQR) M 3
G01-PRMTA	7.0 (4.8–10.8)	6.8 (4.3–8.3)	4.1 (3.2–5.5)
G02-PRMTA-B	11.1 (6.9–12.5)	5.5 (4.7–9.1)	4.7 (3.4–5.2)
G03-MMTA	10.7 (7.1–12.5)	4.9 (3.5–7.4)	4.5 (3.5–6.9)
G04-MMTA-B	10.1 (8.6–11.9)	6.2 (4.9–7.6)	3.8 (3.2–5.4)
G05-TF	9.1 (7.8–12.8)	9.0 (6.2–9.9)	3.3 (2.8–5.5)
G06-TF-B	7.3 (5.9–8.5)	5.7 (4.5–6.5)	4.4 (3.3–5.8)
G07-MPC-Bi	7.7 (5.7–11.9)	7.1 (5.0–8.1)	4.4 (3.8–6.9)
G08-MPC-Bi-B	10.2 (8.3–14.6)	5.8 (3.8–7.6)	4.3 (3.1–5.8)
G09-MPC-Bi-Retro-B	6.5 (4.9–9.8)	7.9 (6.4–10.3)	3.1 (2.4–4.3)
G10-MPC-Bi-Adh-B	8.4 (6.7–9.8)	6.7 (3.8–7.3)	6.9 (4.6–7.9)
All groups pooled	9.1 (6.2–11.4)	6.5 (4.5–8.4)	4.3 (3.1–5.6)

Medians and interquartile ranges of ∆E (∆E*_ab_) were calculated for each group at the specified time points. One-way analysis of variance (ANOVA) with post hoc tests, Friedman test, *t* test, and post hoc tests with Bonferroni correction were performed using IBM SPSS statistics software (Version 24, International Business Machines Corp., Endicott, United States). The normal distribution of the data as a prerequisite for an ANOVA was tested using the Shapiro–Wilk test. The level of significance was set at α = 0.05.

## Results

**Fig. 2:**
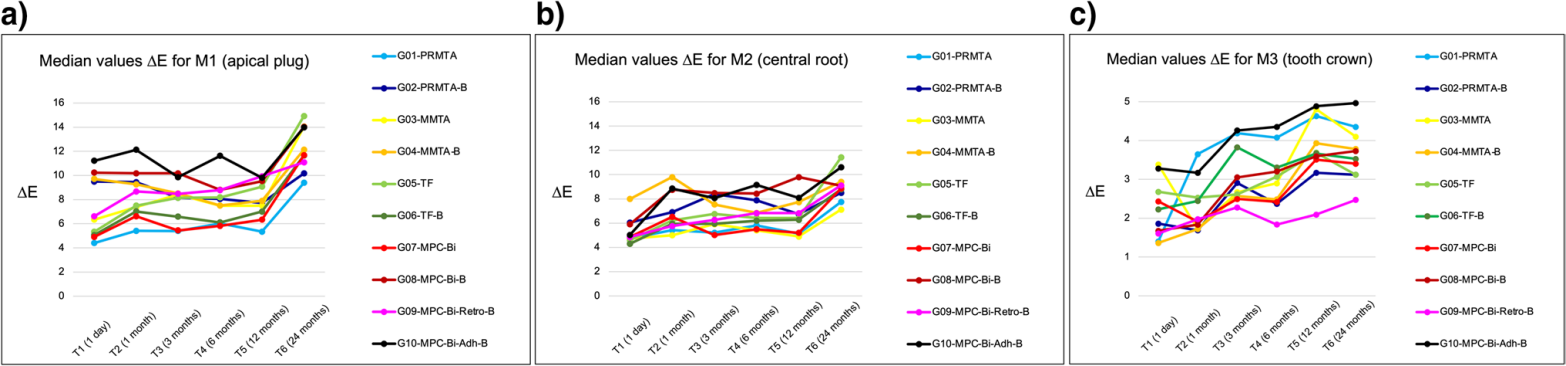
**a** Median color change (∆E) on the apical plug surface (M1) of each group. **b** Median color change (∆E) on the gutta-percha/sealer surface (M2) of each group. **c** Median color change (∆E) on the tooth crown/resin composite surface (M3) of each group

**Fig. 3:**
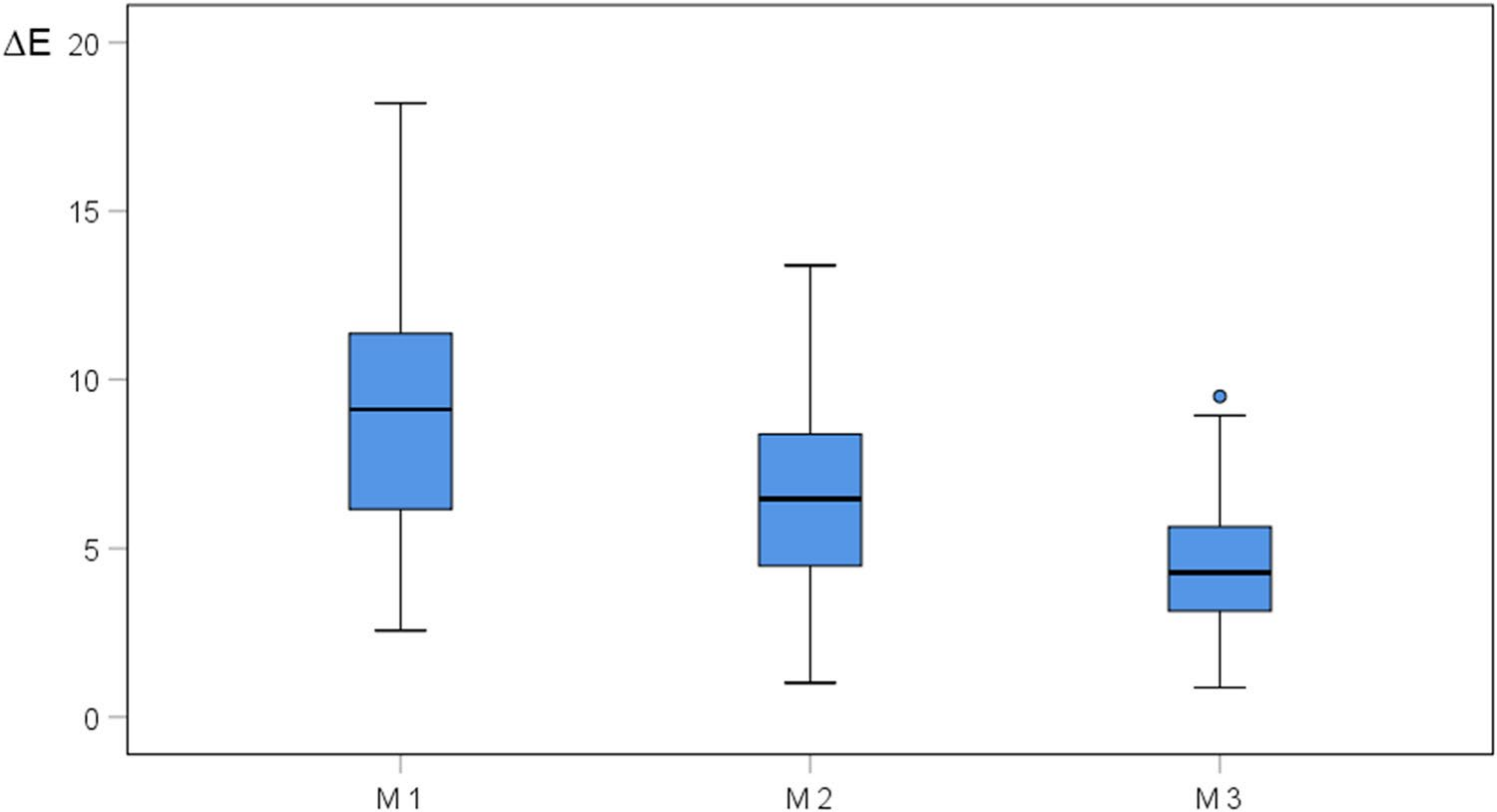
Median color change (∆E) on apical plug (M1), gutta-percha/sealer (M2), and tooth crown/resin composite (M3) surfaces in all groups at 24 months (T6)

All obturation materials had an immediate effect on tooth color: A perceptible color change occurred from before (T0) to 24 h after treatment (T1) on the measuring sites M1 and M2 in all groups (Table 2), not on the measuring site M3. The greatest discoloration was on the apical plug surface followed by the gutta-percha/sealer surface (Tables 2–3). For the next 12 months (T1-T5), ΔE values remained rather at the same level, and these changes were below the perception threshold of human eyesight (Figs. 2a–c). After 24 months (T6), ∆E increased substantially and color differences exceeded the thresholds of human perception (Table 3, Fig. 3). Color differences on all measuring surfaces were statistically significant at 24 months, as determined by post hoc tests with Bonferroni correction (*p* ≤ 0.01). The color difference was then highest on the apical plug and lowest on the tooth crown (Figs. 1c and 3).

Pairwise post hoc tests revealed that there is no significant difference in ∆E on M1 between groups with and without admixture of blood at T6 (*p* > 0.05). However, groups with admixture of blood appeared visually darker than those without blood. Therefore, a detailed analysis of the raw data was performed, whereby the luminescence [L] stood out conspicuously. Specifically, L values were significantly lower in the groups with admixture of blood (post hoc test, *p* < 0.001), which confirmed the visual impression (Table 4).

**Table 4:** Comparison of luminescence [L] on apical plug surfaces (M1) of groups with and without blood (B) contact at 24 months (T6) with standard deviation (SD)

Group	Median L	Mean L	SD	*p*
G01-PRMTA	94.7 (91.4–96.4)	94.0	3.2	.001
G02-PRMTA-B	87.7 (87.0–89.8)	88.0	2.7	
G03-MMTA	97.3 (95.8–98.8)	97.1	2.3	< .001
G04-MMTA-B	89.1 (87.7–89.6)	88.8	2.5	
G05-TF	95.9 (94.0–96.7)	95.4	2.8	.030
G06-TF-B	91.4 (89.1–93.1)	91.1	2.0	
G07-MPC-Bi	93.6 (91.2–94.8)	93.3	2.5	< .001
G08-MPC-Bi-B	86.5 (85.1–87.4)	86.1	2.7	

A retrograde placement (group 9) showed a tendency towards less discoloration of the tooth crown at 24 months than orthograde placement (group 8) (Table 3), but the difference was very small and not statistically significant (*t* test, *p* = 0.160). No effect of prior adhesive dentin sealing of the coronal cavity (group 10 vs. group 8; *p* = 0.123) could be detected at 24 months (Table 3). Overall, the coronal discolorations (M3) were very mild compared with the substantial discolorations of the root (M1 and M2) after 2 years,, but they were mostly still above the perception threshold.

## Discussion

Apical plugs made of HCSC were discussed to cause coronal tooth discoloration, up to 22.7% of teeth following white MTA placement [26], and may therefore compromise aesthetics. This in vitro study was conducted to assess tooth discoloration in the aesthetic relevant zone induced by HCSC-based apical plugs with and without blood contamination. The study was designed to simulate clinical treatment approaches using a standardized rinsing protocol, orthograde root canal obturation with gutta percha and sealer, and retrograde placement of HCSC.

The null hypothesis was rejected: The amount of discoloration on all measuring surfaces did not differ between the HCSC test groups. The admixture of blood during the HCSC setting time significantly influenced tooth color at the apex, as reflected by a significantly higher ΔE values at T1 (after 24 h) on these surfaces. The first research question regarding the presence of blood was negated: 24 h after APT (T1), tooth color in the blood groups distinctly changed due to the external visibility of the materials used for obturation. However, there was no further change in tooth color during the following 12 months. After 2 years, tooth color at the apex where the apical plug was placed changed significantly. But the tooth crown did not show any HCSC-related discoloration.

Neither retrograde placement of the plug nor adhesive preoperative dentin sealing of the access cavity showed any benefit in terms of preventing tooth discoloration. Thus, both the second and the third research questions regarding retrograde placement and preoperative dentin sealing were negated.

In the present investigation bovine teeth of 4 to 6-year-old donor animals were used, and therefore a higher amount of sclerotic dentinal tubules in the sample teeth might have retarded discolorations. Consequently, it can be assumed that stronger discolorations may occur after observation periods of more than 2 years.

One limitation of the present study is that the HCSCs were mixed with blood in all groups aiming to simulate contamination with tissue fluids from the apex, which differs from the clinical situation during placement of the apical barrier. As shown by Ashofteh Yazdi et al. [39] and Son et al. [40] blood contamination can hamper the HCSCs’ setting and chemical properties which might intensify discolorations. Nevertheless, we chose to add a well-defined small amount of blood to better standardise apical contamination and simulate a worst-case scenario. This approach resulted in discoloration of the root but without affecting the visible coronal area.

Furthermore, it can be assumed that the thickness of the dentinal wall plays a crucial role in tooth discoloration. In clinical settings, human teeth with open apices are those requiring root-end-closure with HCSC plugs. These immature teeth typically have thin dentinal walls with wide dentinal tubules. Thus, tooth discoloration may occur faster in clinical situations than in the present study, as is frequently observed in daily dental practice.

A commonly used clinical irrigation protocol (NaOCl and EDTA) for smear layer removal was also performed, as described in a similar study [23]. To exclude interactions between HCSCs, sodium hypochlorite, and Bi2O3, which may exacerbate discoloration [11, 41], the specimens were stored in distilled water for 24 h in an incubator at 37° C after rinsing.

Akbari [27] reported that prior application of dentin bonding agent for adhesive sealing of dentin tubules in the coronal cavity may reduce visible tooth discoloration produced by HCSCs. In their investigation, HCSC plugs were placed up to 1 mm below the CEJ in human teeth. They hypothesized that this was due to a reduction of adhesion of the MTA on the pretreated dentin surface, which facilitates the removal of excess material. In the present study, no benefit of prior dentin sealing on coronal discoloration related to apical plugs could be detected. However, this may be because bovine teeth are larger than human teeth and accommodate larger access cavities. Thus, it was easier to avoid contamination of the coronal dentin walls in the animal model than it might be in the clinical treatment in human teeth.

All four test HCSCs caused tooth discoloration in the apical area during the 24-month observation period, and there were no statistically significant differences between the materials. The most prominent color change appeared 24 h after obturation. This can be attributed to the graying effect of the obturation materials showing through the translucent dental hard tissues [21].

After 24 months (T6), Bi2O3 containing groups without blood (G01-PRMTA and G07-MPC-Bi) showed mild discolorations of the root (M1: median ΔE = 7.0 and 7.7) compared with their corresponding groups with blood (G01-PRMTA-B and G07-MPC-Bi-B) showing severe discolorations (M1: median ΔE = 11.1 and 10.2). However, a HCSC group that does not contain Bi2O3 (G05-TF) showed a higher discoloration of the root (M1: median ΔE = 9.1) compared to when mixed with blood (G05-TF-B; M1: median ΔE = 7.3). Due to the limitations of the present study setup, we could not reveal any discoloration potential of Bi2O3. However, it was concluded that blood has a greater impact on tooth discoloration than Bi2O3 alone, as shown by Dettwiler et al. who revealed that the presence of Bi2O3 was not a guarantor for discolorations [23].

Discoloration occurring with Portland cement may have to do with the incorporation of iron oxide into the tetracalcium alumino-ferrite phase [21] and the oxidation of Bi2O3 [24]. Furthermore, it is reported that the amino acids in collagen destabilize the Bi2O3 molecule, leading to discoloration [14]; this might have had an effect in the present study as well. Other interactions (e.g., HCSC and sodium hypochlorite, light exposure or heat) could be excluded, as mentioned above.

When comparing the groups without blood at 24 months, the severest discoloration was found in group 5 (Total Fill® BC RRM) and the mildest in group 1 (ProRoot® MTA). However, the difference was clinically irrelevant and below the threshold of acceptance. Group 5 (Total Fill® BC RRM), containing zirconium oxide and tantalum pentoxide, showed a greater color change than group 1 (Bi2O3-containing ProRoot® MTA) after 24 months. This confirms previous reports that Bi2O3 does not seem to be a reliable predictor for tooth discoloration [23].

The admixture of bovine blood during the setting time of HCSCs is a clinically relevant condition, which may result in increased tooth staining [12, 13]. Blood components can be taken up by endodontic cements, and subsequent interaction between light and the physiologic degradation of erythrocytes may occur [21]. Within the 24-month observation period of the present study, no HCSC-related discoloration was observed in the aesthetic zone. However, it is possible that it might take longer for potential color changes to develop.

## Conclusions

Apical plugs of the tested HCSCs did not cause discoloration of bovine tooth crowns within a 24-month observation period, if placed carefully while avoiding all direct contact with the coronal dentin. Minor root discoloration was observed. Furthermore, the root canal filling of gutta-percha and sealer must be placed sufficiently below the CEJ followed by adequate composite filling of the coronal cavity. Studies with longer observation periods may be needed to determine if crown discoloration might occur later.
