# Correction to: Tooth discoloration induced by apical plugs with hydraulic calcium silicate-based cements in teeth with open apices—a 2-year in vitro study

**DOI:** 10.1007/s00784-025-06178-8

**Published:** 2025-02-22

**Authors:** Ralf Krug, C. Ortmann, S. Reich, B. Hahn, G. Krastl, S. Soliman

**Affiliations:** 1 Department of Conservative Dentistry and Periodontology and Center of Dental Traumatology, University Hospital of Würzburg, Pleicherwall 2, 97070 Würzburg, Germany; 2 Radolfzell, Germany; 3 Eichstätt, Germany

**Correction to: Clinical Oral Investigations**

10.1007/s00784-021-04009-0

The authors would like to correct Figs. 2b and 2c from the paper.

The original article has been corrected.

